# Supplementary material for: Premature commitment to uncertain decisions during human NMDA receptor hypofunction
Source: Nat Commun. 2022 Jan 17;13:338. doi: 10.1038/s41467-021-27876-3 (PMC8763907; doi:10.1038/s41467-021-27876-3)
Supplement: Supplementary file 3 — Reporting summary [file 41467_2021_27876_MOESM3_ESM.pdf]

nature portfolio

Corresponding author(s): Valentin Wyart
Last updated by author(s): 2021/11/02

Reporting Summary

Nature Portfolio wishes to improve the reproducibility of the work that we publish. This form provides structure for consistency and transparency in reporting. For further information on Nature Portfolio policies, see our Editorial Policies and the Editorial Policy Checklist.

Statistics

For all statistical analyses, confirm that the following items are present in the figure legend, table legend, main text, or Methods section.

n/a	Confirmed	
☐	☒	The exact sample size (*n*) for each experimental group/condition, given as a discrete number and unit of measurement
☐	☒	A statement on whether measurements were taken from distinct samples or whether the same sample was measured repeatedly
☐	☒	The statistical test(s) used AND whether they are one- or two-sided<br>*Only common tests should be described solely by name; describe more complex techniques in the Methods section.*
☐	☒	A description of all covariates tested
☐	☒	A description of any assumptions or corrections, such as tests of normality and adjustment for multiple comparisons
☐	☒	A full description of the statistical parameters including central tendency (e.g. means) or other basic estimates (e.g. regression coefficient) AND variation (e.g. standard deviation) or associated estimates of uncertainty (e.g. confidence intervals)
☐	☒	For null hypothesis testing, the test statistic (e.g. *F*, *t*, *r*) with confidence intervals, effect sizes, degrees of freedom and *P* value noted<br>*Give P values as exact values whenever suitable.*
☐	☒	For Bayesian analysis, information on the choice of priors and Markov chain Monte Carlo settings
☒	☐	For hierarchical and complex designs, identification of the appropriate level for tests and full reporting of outcomes
☐	☒	Estimates of effect sizes (e.g. Cohen's *d*, Pearson's *r*), indicating how they were calculated

Our web collection on statistics for biologists contains articles on many of the points above.

Software and code

Policy information about availability of computer code

Data collection	The cognitive task was coded using the Psychtoolbox-3 toolbox and additional scripts written in MATLAB (version R2019b, The Mathworks). Behavioral responses were collected using a Cedrus response pad (Cedrus Corporation) using functions implemented in Psychtoolbox-3. EEG data were recorded using a 64-channel BioSemi ActiveTwo system (BioSemi, Amsterdam, The Netherlands) at a sampling frequency of 1,024 Hz using the BioSemi ActiView acquisition program for the ActiveTwo system.
Data analysis	Cognitive modeling of the behavioral data was performed using the SPM 12 toolbox for MATLAB, and additional scripts written in MATLAB. The preprocessing of the EEG data was performed using the EEGLAB (http://sccn.ucsd.edu/eeglab, version 13_02_2b) and FieldTrip (http://www.fieldtriptoolbox.org, version 2018/04/04) toolboxes for MATLAB, and additional scripts written in MATLAB. Multivariate pattern analyses of EEG data were performed using scripts written in MATLAB.

For manuscripts utilizing custom algorithms or software that are central to the research but not yet described in published literature, software must be made available to editors and reviewers. We strongly encourage code deposition in a community repository (e.g. GitHub). See the Nature Portfolio guidelines for submitting code & software for further information.

Data

Policy information about availability of data

All manuscripts must include a data availability statement. This statement should provide the following information, where applicable:

- Accession codes, unique identifiers, or web links for publicly available datasets
- A description of any restrictions on data availability
- For clinical datasets or third party data, please ensure that the statement adheres to our policy

The manuscript includes all datasets generated or analyzed during this study. Source data are pro-vided with this paper. Because tested participants did not provide

explicit written consent regarding the posting of their anonymized data on public repositories, individual anonymized behavioral and EEG datasets are available from the corresponding author upon request.

Field-specific reporting

Please select the one below that is the best fit for your research. If you are not sure, read the appropriate sections before making your selection.

- [x] Life sciences
- [ ] Behavioural & social sciences
- [ ] Ecological, evolutionary & environmental sciences

For a reference copy of the document with all sections, see nature.com/documents/nr-reporting-summary-flat.pdf

Life sciences study design

All studies must disclose on these points even when the disclosure is negative.

Sample size	24 adult participants were recruited through public advertisement in the Paris area. The recruited participants were between 18 and 39 years of age, right-handed, reported no history of neurological or psychiatric disease, and no family history of psychotic disorders. Participants reported no addiction to psychoactive drugs, nor history of psychotropic medication. Participants had normal or corrected-to-normal vision, no history of cardiac disease, and blood pressure under 140/90 mmHg. Female participants were not pregnant nor breastfeeding. This sample size is similar to those generally employed for comparable studies (e.g., Vinckier et al., 2016, Molecular Psychiatry).
Data exclusions	The first three participants were used to pilot the different aspects of the study (including parameters of the pharmacological protocol and those of the cue combination task). The EEG data of a fourth participant were lost, and two additional participants did not complete the cue combination task under ketamine as a result of adverse effects.
Replication	A within-subject (crossover) design was employed to account for interindividual variability in EEG and behavioral measures for all analyses reported in the manuscript. A random-effects approach was used in all within-subject statistical analyses to account for interindividual variability in pharmacological effects, and assess the reproducibility of pharmacological effects across tested subjects.
Randomization	The study relies on a double-blind, placebo-controlled, randomized crossover protocol. Each participant performed the cue combination task described below twice, once under ketamine and once under placebo (sodium chloride), during two experimental sessions taking place on separate days. The allocation to receive ketamine (or placebo) on the first session was randomized using a randomization block size of two participants. Both participants and experimenters were blind to this allocation.
Blinding	As indicated above, the allocation to receive ketamine (or placebo) on the first session was randomized using a randomization block size of two participants. Both participants and experimenters were blind to this allocation.

Reporting for specific materials, systems and methods

We require information from authors about some types of materials, experimental systems and methods used in many studies. Here, indicate whether each material, system or method listed is relevant to your study. If you are not sure if a list item applies to your research, read the appropriate section before selecting a response.

Materials & experimental systems

n/a	Involved in the study	
x		Antibodies
x		Eukaryotic cell lines
x		Palaeontology and archaeology
x		Animals and other organisms
	x	Human research participants
x		Clinical data
x		Dual use research of concern

Methods

n/a	Involved in the study	
x		ChIP-seq
x		Flow cytometry
x		MRI-based neuroimaging

Human research participants

Policy information about studies involving human research participants

Population characteristics	The recruited participants were between 18 and 39 years of age, right-handed, reported no history of neurological or psychiatric disease, and no family history of psychotic disorders. Participants reported no addiction to psychoactive drugs, nor history of psychotropic medication. Participants had normal or corrected-to-normal vision, no history of cardiac disease, and blood pressure under 140/90 mmHg. Female participants were not pregnant nor breastfeeding.
Recruitment	The participants were recruited through public advertisement in the Paris area. All were naive to the purpose of the study. As in similar studies conducted in similar conditions (e.g., Vinckier et al., 2016, Molecular Psychiatry), no procedure was used to actively suppress potential self-selection bias in subject recruitement. These biases are unlikely to affect the reported results, given the within-subject design used to analyze the data and the blinding of participants and experimenters to the allocation to receive ketamine or placebo on each session.
Ethics oversight	The study received ethical approval from relevant authorities: the Agence Nationale de Sécurité du Médicament et des Produits de Santé and the Comité de Protection des Personnes Ile-de-France III (ID RCB: 2013-002056-33). The study was registered under reference NCT02235012.

Note that full information on the approval of the study protocol must also be provided in the manuscript.
